# Trends in suicide mortality among cancer survivors in the US, 1975-2020

**DOI:** 10.18632/aging.205451

**Published:** 2024-01-22

**Authors:** Hao Wan, Ru Chen, Xiangpeng Zhan, Luyao Chen, Zhongyuan Li

**Affiliations:** 1 Department of Urology, The First Affiliated Hospital of Nanchang University, Nanchang, Jiangxi Province, China; 2 Senior Department of Pediatrics, The Seventh Medical Center of PLA General Hospital, Beijing, China; 3 Medical School of Chinese PLA, Beijing, China; 4 Department of Urology, Fujian Medical University Union Hospital, Fuzhou, Fujian, China

**Keywords:** suicide mortality, cancer, SEER, trend

## Abstract

Background: Suicide in cancer survivors is a major public health concern, but its trends and risk factors are not well understood. This study aimed to investigate the standardized mortality rate (SMR) and trends in suicide among cancer survivors in the United States.

Methods: Using data from the SEER-9 database and US Mortality data, we identified 3,684,040 cancer survivors diagnosed between 1975 and 2020. The SMR of suicide among cancer survivors was calculated, and Poisson regression analysis was used to evaluate trends in suicide risk. Subgroup analyses were performed based on age, gender, race, tumor site, and stage. A competing risk model was used to calculate the 10-year cumulative incidence of suicide.

Results: Among cancer survivors, the overall SMR of suicide was 1.49 (95%CI: 1.46-1.53) times higher than that of the general population in the US. The risk of suicide varied significantly by cancer site, with the highest risk found in patients with malignant respiratory system cancer. Overall, we observed a significant downward trend in the suicide mortality rate among cancer patients. The cumulative incidence of suicide mortality among cancer survivors across four study periods exhibited significant statistical differences (*P*<0.001).

Conclusions: Our study highlights the need for targeted suicide prevention efforts for cancer survivors, particularly those diagnosed with respiratory system cancer. The trend of declining suicide mortality rates among cancer survivors is promising, but continued efforts are needed to understand and address the underlying risk factors.

## INTRODUCTION

Cancer is a significant global health challenge. It is one of the leading causes of death worldwide and remains a major medical, social, and economic burden. According to the World Health Organization (WHO), cancer is expected to cause 10 million deaths in 2020, with the number projected to increase in the coming years [1]. While cancer-related mortality is primarily attributed to the progression of the disease, cancer survivors also face an increased risk of mortality due to other non-cancer causes. Among these causes, the prevalence of suicide among cancer patients has become a critical public health issue.

Suicide, which refers to the intentional act of ending one’s own life, is a major concern globally and remains one of the leading causes of death, with roughly 800,000 deaths annually, according to the World Health Organization (WHO) [2]. While the causes of suicide are multifaceted, recent research indicates that cancer patients may face a higher risk of suicidal behavior than the general population [3]. A review of 28 studies showed that cancer patients are nearly twice as likely to commit suicide compared to those in the general population, with patients with a poor prognosis being at the highest risk of suicidal behavior. Some of the highest risk groups include patients with esophageal cancer, hepatobiliary cancer, mesothelioma, and pancreatic cancer. Interestingly, suicidal mortality rates were higher in cancer patients in the United States compared to those in Europe, Asia, or Australia, but no such differences were found in the general population [2–4]. Given these findings, screening for suicide and management of risk should be incorporated as basic elements in the guidelines for cancer patient care, pain screening, and management. However, specific suicide prevention strategies for cancer patients remain limited, which poses a challenge for the care of these patients [5].

In this study, we aimed to investigate the prevalence and trends of suicide among cancer survivors using the Surveillance, Epidemiology, and End Results (SEER) database. We intended to provide a comprehensive assessment of the prevalence of suicide among different types of cancer patients and explore the changes in suicide rates over time. This research is necessary to identify the extent of this public health issue and proposes effective prevention strategies for cancer patients at high risk of suicide.

## RESULTS

### Distribution and standardized mortality rate of suicide among cancer survivors

Table 1 displayed the distribution of suicide cases among cancer survivors. Overall, out of 3,684,040 cancer survivors, 7,392 individuals died due to suicide, representing 0.2% of the total cases. Survivors of oral cavity and pharynx cancer had the highest suicide death rate, at 0.45% of the total cases, whereas breast cancer survivors have the lowest suicide death rate at 0.09%. Table 2 presented the SMR for suicide among cancer survivors, as well as subgroup analyses. The data showed that the risk of suicide for all cancer sites was 1.49(95%CI: 1.46-1.53) times higher than that of the general US population, with significant differences between different types of cancer. Patients with malignant respiratory system cancer have the highest SMR, at 3.5 (95%CI: 3.74-4.98), while those with other cancers have the lowest SMR, at 1.1(95%CI: 1.02-1.19) times. In addition, according to a subgroup analysis by age, gender, race, and stage, the results indicated that cancer patients have a significantly higher suicide rate compared to the general population in the United States.

**Table 1:** Distribution of suicide cases among cancer survivors.

	**Death case**	**Suicide cases (n, %)**	**Non-suicide cases (n, %)**
**All sites**	3684040	7392(0.2%)	3676648(99.8%)
**Oral cavity and pharynx**	93,179	422(0.45%)	92757(99.55%)
**Digestive system**	689,513	1171(0.17%)	688342(99.83%)
**Respiratory system**	474,114	871(0.18%)	473,243(99.82%)
**Breast**	560211	523(0.09%)	559688(99.91%)
**Genital system**	807534	2197(0.27%)	805337(99.73%)
**Blood system**	459673	868(0.19%)	458805(99.81%)
**Urinary system**	250908	663(0.26%)	250245(99.74%)
**Other cancer**	348908	677(0.19%)	348231(99.81%)

**Table 2:** Standardized mortality rate of suicide among cancer survivors and subgroup analysis.

	**Observed**	**Expected**	**SMR**	**95%CI**
**All site**	7392	4947	1.49	1.46-1.53
Oral cavity and pharynx	422	149	2.82	2.55-3.1
Digestive system	1171	666	1.76	1.66-1.86
Respiratory system	871	248	3.5	3.74-4.98
Breast	523	388	1.35	1.23-1.47
Genital system	2197	1901	1.16	1.11-1.2
Blood system	868	486	1.78	1.67-1.91
Urinary system	663	492	1.35	1.25-1.45
Other cancer	677	613	1.10	1.02-1.19
**Age(year)**				
<60	2759	2034	1.36	1.31-1.41
60-70	2121	1361	1.56	1.49-1.63
>70	2512	1552	1.62	1.56-1.68
**Gender**				
Male	6037	4014	1.50	1.47-1.54
Female	1355	933	1.45	1.38-1.53
**Race**				
white	6878	4652	1.48	1.44-1.51
black	166	104	1.59	1.36-1.85
others	348	190	1.82	1.64-2.03
**Stage**				
Localized	2152	1748	1.23	1.18- 1.28
Regional	1226	657	1.87	1.76- 1.97
Distant	775	281	2.75	2.56- 2.96
Unknown	2363	1334	1.77	1.7- 1.84
**Tumor number**				
solitary tumor	6504	3770	1.72	1.68- 1.77
multiple tumors	888	1177	0.75	0.71- 0.81

SMR, standardized mortality rate; CI, confidence interval.

### Trends in suicide SMR among cancer survivors by calendar year of diagnosis

**Figure 1:**
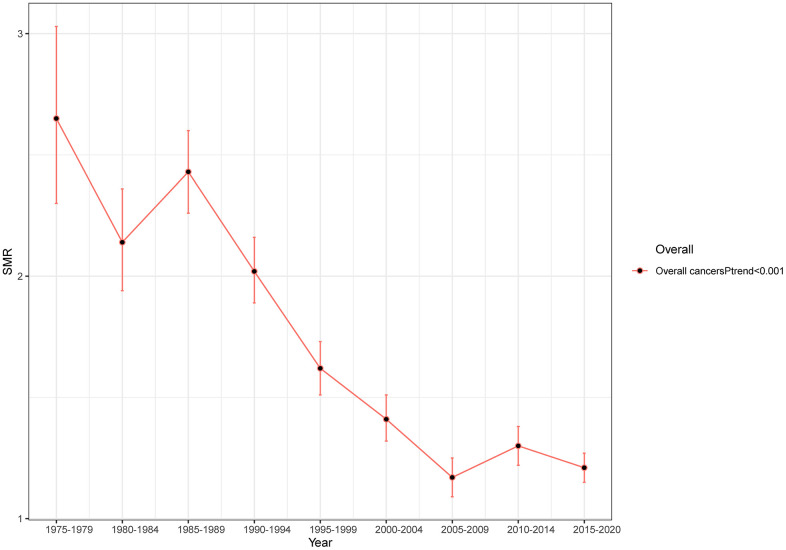
Trends in suicide SMR among all cancer survivors with year of diagnosis.

**Figure 2:**
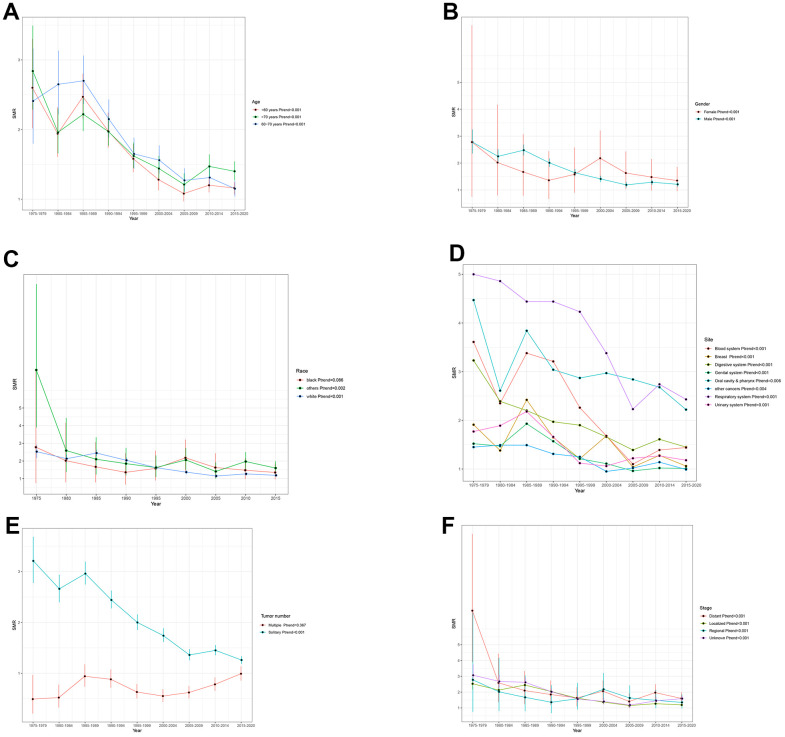
Trends in suicide SMR among all cancer survivors by (**A**) age (**B**) gender (**C**) race (**D**) site (**E**) Tumor number (**F**) stage.

Overall, we could observe a significant downward trend in suicide mortality rates among cancer patients. The SMR, which was 2.65 (95% CI: 2.3-3.03) from 1975-1979, has decreased to 1.21 (95% CI: 1.15-1.27) from 2015-2020(*Ptrend*<0.001) (Figure 1). In sub-group analysis of age, gender, and tumor stage, the suicide mortality rate among cancer patients remains consistent with the overall trend of decline (Figure 2A, 2B, 2F) (all *Ptrend*<0.001). The results of subgroup analysis based on tumor sites also revealed a decreasing trend in the suicide mortality rate for tumors in each location (Figure 2D). It was noteworthy that we did not observe a decreasing trend in suicide death rates among Black cancer survivors (*Ptrend*=0.086) and those with multiple tumors (*Ptrend*=0.367) (Figure 2C, 2E). The suicide SMR among cancer survivors of calendar year of diagnosis were recorded in Supplementary Table 2.

### Cumulative mortality of suicide

**Figure 3:**
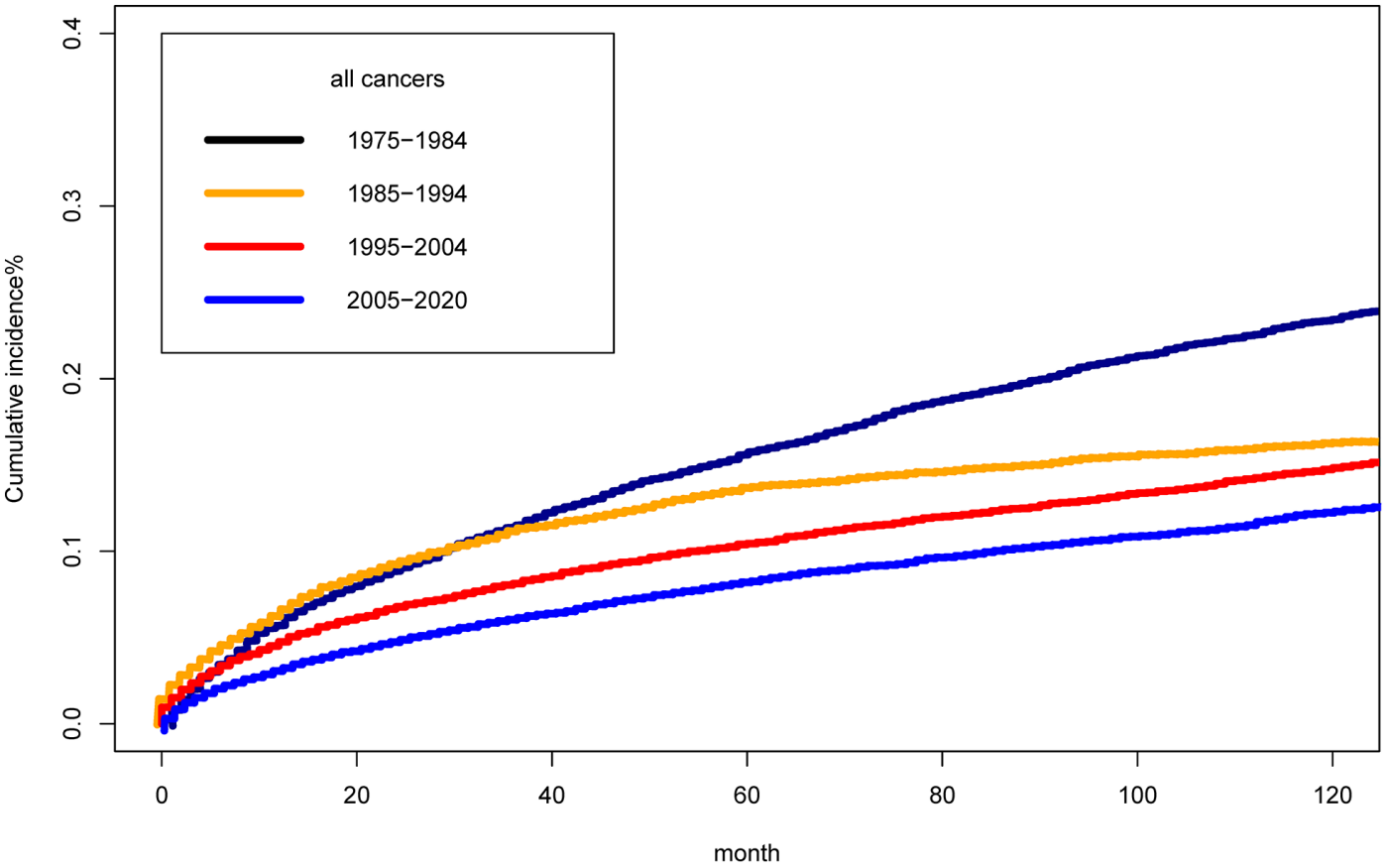
Cumulative mortality for suicide among cancer survivors by year of diagnosis.

Figure 3 presented the clinical burden of suicide differed in the calendar year of diagnosis of cancer survivors based on cumulative suicide mortality analysis. The cumulative incidence of suicide mortality among cancer survivors across four study periods was found to exhibit significant statistical differences (*P*<0.001). The cumulative suicide mortality in 2005-2020 was significantly lower than that in 1975-1984. The 10 years cumulative suicide mortality was 0.22%, 0.14%, 0.11%, 0.09% in period of 1975-1984, 1985-1994, 1995-2004, 2005-2020, respectively.

In subgroup analysis by tumor sites, we could observe that the cumulative suicide incidence rate among eight systemic cancers Supplementary Figure 1 had significant statistical differences in four period (all *P*<0.001). The 10-year cumulative suicide mortality rates for seven system cancers, including oral cavity and pharynx, digestive system, respiratory system, breast, genital system, blood system, and urinary system, were gradually decreasing, besides other cancers.

## DISCUSSION

The present study utilized the SEER database to examine the suicide rates among cancer survivors diagnosed between 1975 and 2020. The results showed that the risk of suicide among cancer survivors was 1.49 times higher than that of the general population in the United States, with significant differences between different types of cancer. Furthermore, we observed a significant downward trend in suicide mortality rates among cancer patients, with the SMR decreasing from 2.65 in 1975-1979 to 1.21 in 2015-2020. The cumulative suicide incidence rate among eight systemic cancers also showed significant statistical differences over time, with a decreasing trend in seven systemic cancers.

Our study highlighted the high risk of suicide among cancer survivors, which should be of concern to healthcare providers and policymakers. The findings are consistent with previous studies, which have reported an increased risk of suicide among cancer patients compared to the general population [2, 3, 6, 7]. However, our study updates and expands on these results by investigating trends in suicide rates among cancer patients over a 45-year period, providing a more comprehensive understanding of this public health issue.

Cancer patients face a myriad of challenges that can increase their vulnerability to suicide. From the moment of diagnosis, they often experience a range of distressing emotions including sadness, anxiety, and depression. The weight of these intense feelings can make it difficult for patients to navigate their cancer journey and can heighten the risk of suicidal thoughts or behaviors.

The physical toll of cancer treatment also contributes to the increased risk. Patients may suffer from severe fatigue and pain, which can significantly decrease their quality of life and further exacerbate feelings of hopelessness and despair. This combination of physical and emotional suffering can leave individuals feeling overwhelmed and increase their risk of suicide [2, 8]. Additionally, cancer treatment can also cause physical symptoms such as fatigue and pain, which can further decrease quality of life and elevate suicidal thoughts [9]. Moreover, cancer survivors may also experience financial difficulties and social isolation, which can exacerbate their distress and contribute to suicidal feelings. They might feel overwhelmed with mounting medical bills, a loss of income, and difficulty performing day-to-day activities. This can lead to a feeling of hopelessness, which can indicate an increased risk of suicide. Lastly, cancer patients might also face stigma and discrimination from society, which can lead to further psychological distress. This can make them feel unworthy and unimportant and can further amplify suicidal thoughts. Overall, cancer patients require adequate psychological support and care to help them cope with their diagnosis, treatment, and recovery and reduce their risk of suicide [10, 11].

This study makes a more intriguing finding, namely, the significant decreasing trend in suicide mortality rates among U.S. cancer patients over the last 45 years. There are several factors that may have contributed to the result. One significant factor is the marked improvement in cancer treatment over the past few decades, resulting in better cancer survival rates. This improved prognosis may have positively impacted the mental health of cancer patients, ultimately lowering the risk of suicidal behavior [1, 5]. Another likely contributor is the advancements in psychosocial support and interventions for cancer patients [12]. Nowadays, cancer patients have access to a range of therapies such as counseling, group therapy, and pharmacotherapy. These treatment options not only address the physical aspects of cancer but also the psychological and emotional toll that the disease can take on patients and their families. This increased support may have contributed to the decreasing trend in suicide rates among cancer patients [13, 14]. Lastly, the growing awareness and stigma reduction efforts surrounding mental health and suicide in the general population may have also positively impacted cancer patients. Increased public education and awareness of issues related to mental health and suicide may have helped to mitigate some of the negative stigmas associated with seeking help for mental health issues. This awareness may have trickled down to cancer patients, leading to a decrease in suicidal ideation and behavior [15, 16].

Our findings have important implications for clinical practice, suggesting that mental health support and interventions may improve the quality of life and survival outcomes for cancer patients. It is crucial for healthcare providers to assess and address mental health concerns among cancer patients, particularly those at higher risk for suicidal behavior. Future research should focus on identifying effective interventions to reduce the risk of suicidal behavior among cancer patients, particularly for those at highest risk.

Despite the strengths of this study, there are several limitations that should be acknowledged. First, the study was limited to cancer patients diagnosed and treated at SEER-8 sites, and therefore, may not be representative of the broader population of cancer patients in the US. Second, the study relied on administrative data, which may be subject to errors and biases. Third, the study was unable to account for potential confounding factors, such as co-morbid mental health conditions or social determinants of health, which may impact the risk of suicidal behavior among cancer patients. Lastly, we classified all cancers in a systematic manner, rather than specifically analyzing each individual cancer type. This approach may have limitations, as the number of suicide deaths among individual cancer types is relatively small. As a result, our findings may have more limited generalizability.

## CONCLUSIONS

In conclusion, our study provides evidence of a decreasing trend in suicide mortality rates among cancer patients over the past few decades. Further research is needed to identify effective interventions to reduce the risk of suicidal behavior among cancer patients, and to mitigate the impact of mental health issues on cancer outcomes.

## MATERIALS AND METHODS

### Data source

This study utilized the SEER-9 database for cancer survivors and a reference cohort from the US Mortality data to investigate suicide trends among cancer patients. The SEER-9 registries cover nine areas across the USA, representing approximately 9.4% of the US population. This database leverages cancer registry data and contains information on cancer incidence, treatment, and survival. The US Mortality Data, maintained by the National Center for Health Statistics (NCHS) of the Centers for Disease Control and Prevention (CDC), were used to determine causes of death and population data based on death certificates. The use of deidentified existing data was approved for exemption from Institutional Review Board by the National Institutes of Health Office of Human Subjects Research. Therefore, the data source for this study included existing, deidentified data from the SEER-9 database and the US Mortality data, with no overlap in participants with the prior prostate cancer study.

It is worth noting that this research was exempt from Institutional Review Board by the National Institutes of Health Office of Human Subjects Research based on the usage of deidentified existing data. With these reliable and comprehensive sources of data, this study was able to draw meaningful conclusions about the survival outcomes of cancer survivors.

### Study population

This study leveraged the expansive and reliable surveillance data of the SEER database, to explore the suicide rates among all cancer patients diagnosed between January 1, 1975 to December 31, 2020. Specifically, a total of 4482414 patients with a malignant tumor were identified, utilizing data from SEER-8 registrations in Atlanta, Utah, Seattle, New Mexico, Iowa, Hawaii, Connecticut, and San Francisco-Oakland SMSA. Patients were excluded based on criteria including: diagnosis age less than 20, unknown survival time, or cause of death. Collected data encompassed important variables such as age at diagnosis, race, year of diagnosis, tumor stage, number of tumors, cause of death, and survival month. All patients included in this study population had known information on suicide and survival time, and were diagnosed and treated at SEER-8 sites.

### Cancer sites

To facilitate analysis, we have classified all types of cancer into eight categories based on their specific sites or regions, including oral cavity and pharynx, digestive system, respiratory system, breast, genital system, blood system, urinary system and other cancer. The more detailed classification is recorded in Supplementary Table 1.

### Suicide mortality and follow-up

The SEER program utilized the International Classification of Diseases (ICD) version 9 from 1979 to 1998 and version 10 from 1998 onwards to record the cause of death (COD) of patients. Suicide cases were coded as E950-E959 based on ICD-9 and U03, X60-X84, and Y87.0 based on ICD-10. To simplify the analysis, we categorized the causes of death of all cancer patients into suicidal and non-suicidal causes. The observation period of this study began at the time of cancer diagnosis and continued until the patient’s death, last recorded as alive, or last follow-up (December 31, 2020), whichever occurred first. The smallest unit of follow-up time was one month.

### Statistical analyses

To investigate the potential difference in suicide risk between cancer survivors and the general population, specifically, we will calculate the standardized mortality rate (SMR) by dividing the observed number of suicide deaths among the cancer survivor cohort by the expected number in the US general population, adjusted for factors such as age at death (5-year groups), race (White/Black/other), and year of death. The number of expected deaths was calculated by multiplying the suicide mortality rate of the general men population in the U.S. by the cumulative follow-up time in the study cohort. The SMR were calculated with SEER*Stat version 8.3.8.

We utilized a Poisson regression model to evaluate the trend of SMR for cancer patients across calendar years (as a continuous variable) with expected events as offset. To test the stability of our findings, sensitivity analyses were conducted by performing subgroup analyses based on age (<60,60-70,>70), sex (male, female), race (white, black, others), cancer site, tumor number (solitary tumor, multiple tumors), and cancer stage (localized, regional, distant). The statistical results were presented as coefficients and *Ptrend*, which respectively represent the direction and significance of the Poisson regression model. To evaluate the clinical burden of suicide death rates, we used a competing risk model to calculate the cumulative suicide death rates (as well as corresponding 95% confidence intervals), while accounting for competing death risks from non-suicidal causes. We calculated 10-year cumulative suicide death rates for cancer patients diagnosed in the calendar year. The competing risk model was performed using R and RStudio version 4.6.0.0 (with the cmprsk package), and the Ptrend test was conducted in Stata version 14 (StataCorp, College Station, TX, USA).

### Availability of data and materials

The data in this article comes from the SEER database. This data can be found here: https://seer.cancer.gov/data-software/documentation/seerstat/nov2020/.

## Supplementary Material

Supplementary Figure 1

Supplementary Table 1

Supplementary Table 2
