# Supplementary material for: Trends in suicide mortality among cancer survivors in the US, 1975-2020
Source: Aging (Albany NY). 2024 Jan 22;16(2):1685–95. doi: 10.18632/aging.205451 (PMC10866445; doi:10.18632/aging.205451)
Supplement: Supplementary Figure 1 [file aging-16-205451-s001.pdf]

SUPPLEMENTARY FIGURE

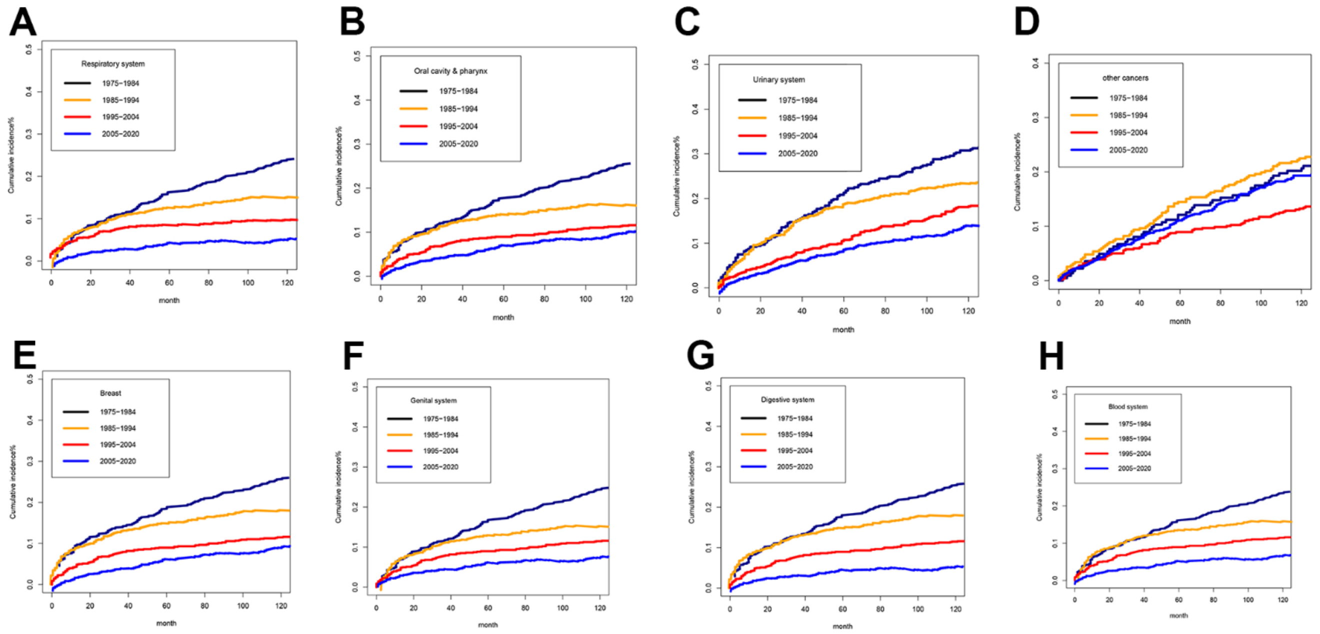

Supplementary Figure 1. Cumulative mortality for suicide among eight system cancer survivors (**A**) Respiratory system, (**B**) Oral cavity and pharynx, (**C**) Urinary system, (**D**) Other cancer, (**E**) Breast, (**F**) Genital system, (**G**) Digestive system, (**H**) Blood system by year of diagnosis.
