# Supplementary material for: Trends in suicide mortality among cancer survivors in the US, 1975-2020
Source: Aging (Albany NY). 2024 Jan 22;16(2):1685–95. doi: 10.18632/aging.205451 (PMC10866445; doi:10.18632/aging.205451)
Supplement: Supplementary Table 1 [file aging-16-205451-s002.pdf]

SUPPLEMENTARY TABLE

Supplementary Table 1. Eight categories of cancers and the list of cancers included in each category.

Oral cavity and pharynx	Tongue, mouth, pharynx, other oral cavity
**Digestive system**	Esophagus, Stomach, Small intestine, Colon, Rectum, Anus, anal canal, and anorectum, Liver and intrahepatic bile duct, Gallbladder and other biliary, Pancreas, Other digestive organs
**Respiratory system**	Larynx, Lung and bronchus, Other respiratory organs
**Breast**	Breast
**Genital system**	Uterine cervix, Uterine corpus, Ovary, Vulva, Vagina and other genital, female, Prostate, Testis, Penis and other genital, male,
**Blood system**	Lymphoma, Myeloma, Leukemia,
**Urinary system**	
**Other cancer**	Bones and joints, Soft tissue (including heart), Skin (excluding basal and squamous), Eye and orbit Brain and other nervous system, Endocrine system, Other and unspecified primary site.
