# Supplementary material for: Trends in suicide mortality among cancer survivors in the US, 1975-2020
Source: Aging (Albany NY). 2024 Jan 22;16(2):1685–95. doi: 10.18632/aging.205451 (PMC10866445; doi:10.18632/aging.205451)
Supplement: Supplementary Table 2 [file aging-16-205451-s003.docx]

| **Supplementary Table 2. Suicide SMR among cancer survivors by calendar year of diagnosis.** | | | | | |
| --- | --- | --- | --- | --- | --- |
| Attained Calendar Year | Observed | Expected | SMR | CI Lower | CI Upper |
| All site | | | | | |
| 1975-1979 | 0 | 79 | 2.65 | 2.3 | 3.03 |
| 1980-1984 | 410 | 21192 | 2.14 | 1.94 | 2.36 |
| 1985-1989 | 788 | 324 | 2.43 | 2.26 | 2.6 |
| 1990-1994 | 874 | 433 | 2.02 | 1.89 | 2.16 |
| 1995-1999 | 810 | 501 | 1.62 | 1.51 | 1.73 |
| 2000-2004 | 807 | 571 | 1.41 | 1.32 | 1.51 |
| 2005-2009 | 812 | 695 | 1.17 | 1.09 | 1.25 |
| 2010-2014 | 1149 | 885 | 1.3 | 1.22 | 1.38 |
| 2015-2020 | 1532 | 1268 | 1.21 | 1.15 | 1.27 |
| Oral cavity & pharynx |  |  |  |  |  |
| 1975-1979 | 17 | 4 | 4.47 | 2.6 | 7.15 |
| 1980-1984 | 23 | 9 | 2.61 | 1.65 | 3.92 |
| 1985-1989 | 51 | 13 | 3.84 | 2.86 | 5.05 |
| 1990-1994 | 45 | 15 | 3.04 | 2.22 | 4.07 |
| 1995-1999 | 43 | 15 | 2.87 | 2.08 | 3.87 |
| 2000-2004 | 46 | 15 | 2.97 | 2.17 | 3.96 |
| 2005-2009 | 52 | 18 | 2.84 | 2.12 | 3.73 |
| 2010-2014 | 64 | 24 | 2.68 | 2.07 | 3.43 |
| 2015-2020 | 81 | 36 | 2.22 | 1.76 | 2.76 |
| Digestive system |  |  |  |  |  |
| 1975-1979 | 50 | 15 | 3.23 | 2.39 | 4.25 |
| 1980-1984 | 85 | 35 | 2.39 | 1.91 | 2.96 |
| 1985-1989 | 132 | 60 | 2.2 | 1.84 | 2.61 |
| 1990-1994 | 143 | 72 | 1.97 | 1.66 | 2.33 |
| 1995-1999 | 142 | 74 | 1.9 | 1.6 | 2.24 |
| 2000-2004 | 129 | 78 | 1.66 | 1.39 | 1.98 |
| 2005-2009 | 120 | 86 | 1.39 | 1.15 | 1.66 |
| 2010-2014 | 165 | 102 | 1.61 | 1.37 | 1.87 |
| 2015-2020 | 205 | 142 | 1.45 | 1.26 | 1.66 |
| Respiratory system |  |  |  |  |  |
| 1975-1979 | 50 | 16 | 3.23 | 2.39 | 4.25 |
| 1980-1984 | 85 | 36 | 2.39 | 1.91 | 2.96 |
| 1985-1989 | 132 | 60 | 2.2 | 1.84 | 2.61 |
| 1990-1994 | 143 | 72 | 1.97 | 1.66 | 2.33 |
| 1995-1999 | 142 | 75 | 1.9 | 1.6 | 2.24 |
| 2000-2004 | 129 | 78 | 1.66 | 1.39 | 1.98 |
| 2005-2009 | 120 | 86 | 1.39 | 1.15 | 1.66 |
| 2010-2014 | 165 | 103 | 1.61 | 1.37 | 1.87 |
| 2015-2020 | 205 | 142 | 1.45 | 1.26 | 1.66 |
| Breast |  |  |  |  |  |
| 1975-1979 | 14 | 7 | 1.91 | 1.05 | 3.21 |
| 1980-1984 | 23 | 17 | 1.38 | 0.88 | 2.07 |
| 1985-1989 | 62 | 26 | 2.42 | 1.85 | 3.1 |
| 1990-1994 | 53 | 32 | 1.65 | 1.24 | 2.16 |
| 1995-1999 | 44 | 36 | 1.21 | 0.88 | 1.62 |
| 2000-2004 | 72 | 43 | 1.68 | 1.31 | 2.11 |
| 2005-2009 | 57 | 55 | 1.04 | 0.78 | 1.34 |
| 2010-2014 | 90 | 70 | 1.28 | 1.03 | 1.57 |
| 2015-2020 | 108 | 102 | 1.06 | 0.87 | 1.28 |
| Genital system |  |  |  |  |  |
| 1975-1979 | 34 | 22 | 1.52 | 1.05 | 2.13 |
| 1980-1984 | 85 | 58 | 1.47 | 1.18 | 1.82 |
| 1985-1989 | 199 | 103 | 1.93 | 1.67 | 2.21 |
| 1990-1994 | 251 | 159 | 1.57 | 1.39 | 1.78 |
| 1995-1999 | 244 | 201 | 1.21 | 1.06 | 1.37 |
| 2000-2004 | 261 | 235 | 1.11 | 0.98 | 1.25 |
| 2005-2009 | 271 | 282 | 0.96 | 0.85 | 1.08 |
| 2010-2014 | 362 | 353 | 1.02 | 0.92 | 1.14 |
| 2015-2020 | 490 | 487 | 1.01 | 0.92 | 1.1 |
| Blood system |  |  |  |  |  |
| 1975-1979 | 26 | 7 | 3.61 | 2.36 | 5.29 |
| 1980-1984 | 39 | 17 | 2.35 | 1.67 | 3.21 |
| 1985-1989 | 95 | 28 | 3.38 | 2.74 | 4.14 |
| 1990-1994 | 117 | 36 | 3.21 | 2.66 | 3.85 |
| 1995-1999 | 93 | 41 | 2.26 | 1.82 | 2.76 |
| 2000-2004 | 84 | 50 | 1.68 | 1.34 | 2.08 |
| 2005-2009 | 76 | 69 | 1.1 | 0.87 | 1.38 |
| 2010-2014 | 131 | 94 | 1.39 | 1.16 | 1.65 |
| 2015-2020 | 207 | 144 | 1.44 | 1.25 | 1.65 |
| **Urinary system** |  |  |  |  |  |
| 1975-1979 | 15 | 8 | 1.77 | 0.99 | 2.91 |
| 1980-1984 | 42 | 22 | 1.89 | 1.36 | 2.55 |
| 1985-1989 | 85 | 39 | 2.18 | 1.74 | 2.7 |
| 1990-1994 | 81 | 49 | 1.66 | 1.32 | 2.07 |
| 1995-1999 | 59 | 53 | 1.12 | 0.85 | 1.44 |
| 2000-2004 | 60 | 57 | 1.06 | 0.81 | 1.36 |
| 2005-2009 | 80 | 66 | 1.22 | 0.97 | 1.52 |
| 2010-2014 | 103 | 81 | 1.27 | 1.03 | 1.53 |
| 2015-2020 | 138 | 117 | 1.18 | 0.99 | 1.39 |
| **Other cancer** |  |  |  |  |  |
| 1975-1979 | 8 | 6 | 1.45 | 0.63 | 2.86 |
| 1980-1984 | 23 | 15 | 1.49 | 0.94 | 2.24 |
| 1985-1989 | 41 | 27 | 1.49 | 1.07 | 2.02 |
| 1990-1994 | 51 | 39 | 1.31 | 0.98 | 1.73 |
| 1995-1999 | 63 | 50 | 1.25 | 0.96 | 1.6 |
| 2000-2004 | 62 | 66 | 0.95 | 0.72 | 1.21 |
| 2005-2009 | 92 | 90 | 1.02 | 0.82 | 1.25 |
| 2010-2014 | 144 | 126 | 1.14 | 0.96 | 1.35 |
| 2015-2020 | 193 | 194 | 0.99 | 0.86 | 1.14 |
| Age<60 year |  |  |  |  |  |
| 1975-1979 | 68 | 26 | 2.6 | 2.02 | 3.3 |
| 1980-1984 | 121 | 62 | 1.94 | 1.61 | 2.32 |
| 1985-1989 | 238 | 96 | 2.47 | 2.16 | 2.8 |
| 1990-1994 | 251 | 127 | 1.98 | 1.74 | 2.24 |
| 1995-1999 | 253 | 160 | 1.58 | 1.39 | 1.79 |
| 2000-2004 | 276 | 216 | 1.28 | 1.13 | 1.44 |
| 2005-2009 | 333 | 308 | 1.08 | 0.97 | 1.2 |
| 2010-2014 | 508 | 424 | 1.2 | 1.1 | 1.31 |
| 2015-2020 | 711 | 615 | 1.16 | 1.07 | 1.24 |
| Age 60-70 year |  |  |  |  |  |
| 1975-1979 | 52 | 22 | 2.41 | 1.8 | 3.16 |
| 1980-1984 | 138 | 52 | 2.65 | 2.22 | 3.13 |
| 1985-1989 | 248 | 92 | 2.7 | 2.37 | 3.06 |
| 1990-1994 | 254 | 118 | 2.15 | 1.89 | 2.43 |
| 1995-1999 | 226 | 137 | 1.65 | 1.44 | 1.88 |
| 2000-2004 | 237 | 152 | 1.56 | 1.37 | 1.77 |
| 2005-2009 | 231 | 182 | 1.27 | 1.11 | 1.44 |
| 2010-2014 | 316 | 241 | 1.31 | 1.17 | 1.47 |
| 2015-2020 | 419 | 366 | 1.15 | 1.04 | 1.26 |
| Age >70 year |  |  |  |  |  |
| 1975-1979 | 90 | 32 | 2.84 | 2.29 | 3.49 |
| 1980-1984 | 151 | 77 | 1.96 | 1.66 | 2.3 |
| 1985-1989 | 302 | 136 | 2.22 | 1.98 | 2.48 |
| 1990-1994 | 369 | 188 | 1.97 | 1.77 | 2.18 |
| 1995-1999 | 331 | 204 | 1.62 | 1.45 | 1.81 |
| 2000-2004 | 294 | 204 | 1.44 | 1.28 | 1.62 |
| 2005-2009 | 248 | 205 | 1.21 | 1.06 | 1.37 |
| 2010-2014 | 325 | 220 | 1.47 | 1.32 | 1.64 |
| 2015-2020 | 402 | 287 | 1.4 | 1.27 | 1.54 |
| White |  |  |  |  |  |
| 1975-1979 | 192 | 76 | 2.53 | 2.18 | 2.91 |
| 1980-1984 | 390 | 183 | 2.13 | 1.92 | 2.35 |
| 1985-1989 | 760 | 310 | 2.45 | 2.28 | 2.63 |
| 1990-1994 | 838 | 411 | 2.04 | 1.9 | 2.18 |
| 1995-1999 | 762 | 471 | 1.62 | 1.5 | 1.74 |
| 2000-2004 | 732 | 536 | 1.37 | 1.27 | 1.47 |
| 2005-2009 | 747 | 651 | 1.15 | 1.07 | 1.23 |
| 2010-2014 | 1051 | 830 | 1.27 | 1.19 | 1.34 |
| 2015-2020 | 1406 | 1184 | 1.19 | 1.13 | 1.25 |
| Black |  |  |  |  |  |
| 1975-1979 | 4 | 1 | 2.78 | 0.76 | 7.11 |
| 1980-1984 | 7 | 3 | 2.02 | 0.81 | 4.16 |
| 1985-1989 | 10 | 6 | 1.67 | 0.8 | 3.06 |
| 1990-1994 | 11 | 8 | 1.36 | 0.68 | 2.43 |
| 1995-1999 | 16 | 10 | 1.58 | 0.91 | 2.57 |
| 2000-2004 | 26 | 12 | 2.18 | 1.43 | 3.2 |
| 2005-2009 | 24 | 15 | 1.63 | 1.04 | 2.42 |
| 2010-2014 | 28 | 19 | 1.48 | 0.99 | 2.14 |
| 2015-2020 | 40 | 30 | 1.35 | 0.97 | 1.84 |
| Other race |  |  |  |  |  |
| 1975-1979 | 14 | 2 | 7.15 | 3.91 | 12 |
| 1980-1984 | 13 | 5 | 2.59 | 1.38 | 4.43 |
| 1985-1989 | 18 | 8 | 2.1 | 1.25 | 3.33 |
| 1990-1994 | 25 | 13 | 1.85 | 1.2 | 2.73 |
| 1995-1999 | 32 | 20 | 1.63 | 1.11 | 2.3 |
| 2000-2004 | 49 | 24 | 2.06 | 1.52 | 2.72 |
| 2005-2009 | 41 | 29 | 1.41 | 1.01 | 1.91 |
| 2010-2014 | 70 | 36 | 1.97 | 1.54 | 2.49 |
| 2015-2020 | 86 | 54 | 1.6 | 1.28 | 1.98 |
| Male |  |  |  |  |  |
| 1975-1979 | 165 | 59 | 2.78 | 2.37 | 3.24 |
| 1980-1984 | 333 | 148 | 2.25 | 2.02 | 2.51 |
| 1985-1989 | 644 | 260 | 2.48 | 2.29 | 2.68 |
| 1990-1994 | 714 | 355 | 2.01 | 1.87 | 2.16 |
| 1995-1999 | 682 | 415 | 1.64 | 1.52 | 1.77 |
| 2000-2004 | 666 | 473 | 1.41 | 1.3 | 1.52 |
| 2005-2009 | 676 | 567 | 1.19 | 1.1 | 1.29 |
| 2010-2014 | 921 | 717 | 1.29 | 1.2 | 1.37 |
| 2015-2020 | 1236 | 1021 | 1.21 | 1.14 | 1.28 |
| Female |  |  |  |  |  |
| 1975-1979 | 45 | 20 | 2.25 | 1.64 | 3.01 |
| 1980-1984 | 77 | 44 | 1.77 | 1.39 | 2.21 |
| 1985-1989 | 144 | 64 | 2.23 | 1.88 | 2.63 |
| 1990-1994 | 160 | 78 | 2.06 | 1.76 | 2.41 |
| 1995-1999 | 128 | 85 | 1.5 | 1.25 | 1.78 |
| 2000-2004 | 141 | 99 | 1.43 | 1.2 | 1.69 |
| 2005-2009 | 136 | 128 | 1.06 | 0.89 | 1.25 |
| 2010-2014 | 228 | 168 | 1.36 | 1.19 | 1.54 |
| 2015-2020 | 296 | 247 | 1.2 | 1.07 | 1.34 |
| Localized |  |  |  |  |  |
| 1975-1979 | 43 | 27 | 1.61 | 1.17 | 2.17 |
| 1980-1984 | 96 | 70 | 1.37 | 1.11 | 1.68 |
| 1985-1989 | 200 | 122 | 1.64 | 1.42 | 1.89 |
| 1990-1994 | 248 | 160 | 1.55 | 1.37 | 1.76 |
| 1995-1999 | 223 | 182 | 1.23 | 1.07 | 1.4 |
| 2000-2004 | 251 | 207 | 1.21 | 1.07 | 1.37 |
| 2005-2009 | 265 | 253 | 1.05 | 0.93 | 1.18 |
| 2010-2014 | 402 | 322 | 1.25 | 1.13 | 1.38 |
| 2015-2020 | 424 | 406 | 1.04 | 0.95 | 1.15 |
| Regional |  |  |  |  |  |
| 1975-1979 | 42 | 14 | 2.96 | 2.14 | 4 |
| 1980-1984 | 62 | 33 | 1.9 | 1.46 | 2.44 |
| 1985-1989 | 159 | 53 | 3.03 | 2.58 | 3.54 |
| 1990-1994 | 154 | 65 | 2.36 | 2 | 2.76 |
| 1995-1999 | 148 | 70 | 2.1 | 1.78 | 2.47 |
| 2000-2004 | 148 | 78 | 1.9 | 1.6 | 2.23 |
| 2005-2009 | 139 | 92 | 1.51 | 1.27 | 1.78 |
| 2010-2014 | 189 | 115 | 1.65 | 1.42 | 1.9 |
| 2015-2020 | 185 | 137 | 1.35 | 1.16 | 1.55 |
| Distant |  |  |  |  |  |
| 1975-1979 | 27 | 7 | 4.07 | 2.68 | 5.92 |
| 1980-1984 | 46 | 12 | 3.74 | 2.74 | 4.99 |
| 1985-1989 | 84 | 18 | 4.58 | 3.66 | 5.67 |
| 1990-1994 | 100 | 23 | 4.28 | 3.48 | 5.2 |
| 1995-1999 | 109 | 27 | 4.06 | 3.34 | 4.9 |
| 2000-2004 | 101 | 32 | 3.17 | 2.58 | 3.85 |
| 2005-2009 | 89 | 41 | 2.16 | 1.74 | 2.66 |
| 2010-2014 | 124 | 56 | 2.22 | 1.84 | 2.64 |
| 2015-2020 | 95 | 65 | 1.46 | 1.18 | 1.79 |
| Solitary tumor |  |  |  |  |  |
| 1975-1979 | 202 | 63 | 3.21 | 2.78 | 3.68 |
| 1980-1984 | 386 | 145 | 2.66 | 2.4 | 2.93 |
| 1985-1989 | 708 | 239 | 2.96 | 2.75 | 3.19 |
| 1990-1994 | 770 | 315 | 2.44 | 2.28 | 2.62 |
| 1995-1999 | 722 | 361 | 2 | 1.86 | 2.15 |
| 2000-2004 | 719 | 412 | 1.74 | 1.62 | 1.88 |
| 2005-2009 | 700 | 514 | 1.36 | 1.26 | 1.47 |
| 2010-2014 | 991 | 682 | 1.45 | 1.36 | 1.55 |
| 2015-2020 | 1306 | 1039 | 1.26 | 1.19 | 1.33 |
| multiple tumors |  |  |  |  |  |
| 1975-1979 | 8 | 16 | 0.49 | 0.21 | 0.96 |
| 1980-1984 | 24 | 46 | 0.52 | 0.33 | 0.77 |
| 1985-1989 | 80 | 85 | 0.94 | 0.74 | 1.17 |
| 1990-1994 | 104 | 118 | 0.88 | 0.72 | 1.07 |
| 1995-1999 | 88 | 140 | 0.63 | 0.51 | 0.78 |
| 2000-2004 | 88 | 159 | 0.55 | 0.44 | 0.68 |
| 2005-2009 | 112 | 181 | 0.62 | 0.51 | 0.74 |
| 2010-2014 | 158 | 203 | 0.78 | 0.66 | 0.91 |
| 2015-2020 | 226 | 229 | 0.99 | 0.86 | 1.13 |
| SMR: standardized mortality rate; CI: confidence interval | | | | | |
